# Silver-nanoparticles-modified biomaterial surface resistant to *staphylococcus*: new insight into the antimicrobial action of silver

**DOI:** 10.1038/srep32699

**Published:** 2016-09-07

**Authors:** Jiaxing Wang, Jinhua Li, Geyong Guo, Qiaojie Wang, Jin Tang, Yaochao Zhao, Hui Qin, Tuerhongjiang Wahafu, Hao Shen, Xuanyong Liu, Xianlong Zhang

**Affiliations:** 1 Department of Orthopaedics, Shanghai Jiao Tong University Affiliated Sixth People’s Hospital, Shanghai Jiao Tong University, Shanghai 200233, China; 2 State Key Laboratory of High Performance Ceramics and Superfine Microstructure, Shanghai Institute of Ceramics, Chinese Academy of Sciences, Shanghai 200050, China; 3 University of Chinese Academy of Sciences, Beijing 100049, China; 4 Department of Clinical Laboratory, Shanghai Jiao Tong University Affiliated Sixth People’s Hospital, Shanghai Jiao Tong University, Shanghai 200233, China

## Abstract

Titanium implants are widely used clinically, but postoperative implant infection remains a potential severe complication. The purpose of this study was to investigate the antibacterial activity of nano-silver(Ag)-functionalized Ti surfaces against epidemic *Staphylococcus* from the perspective of the regulation of biofilm-related genes and based on a bacteria-cell co-culture study. To achieve this goal, two representative epidemic *Staphylococcus* strains, *Staphylococcus epidermidis* (*S. epidermidis*, RP62A) and *Staphylococcus aureus* (*S. aureus*, USA 300), were used, and it was found that an Ag-nanoparticle-modified Ti surface could regulate the expression levels of biofilm-related genes (*ica*A and *ica*R for *S. epidermidis*; *fnb*A and *fnb*B for *S. aureus*) to inhibit bacterial adhesion and biofilm formation. Moreover, a novel bacteria-fibroblast co-culture study revealed that the incorporation of Ag nanoparticles on such a surface can help mammalian cells to survive, adhere and spread more successfully than *Staphylococcus*. Therefore, the modified surface was demonstrated to possess a good anti-infective capability against both sessile bacteria and planktonic bacteria through synergy between the effects of Ag nanoparticles and ion release. This work provides new insight into the antimicrobial action and mechanism of Ag-nanoparticle-functionalized Ti surfaces with bacteria-killing and cell-assisting capabilities and paves the way towards better satisfying the clinical needs.

Commercial titanium (Ti) and titanium alloys have been utilized as biomedical implants for many years, on account of their intrinsic biocompatibility, prominent mechanical properties and good anti-corrosive quality^1^. However, Ti-based implants exhibit poor biological activity, resulting in insufficient osseointegration^1^; moreover, they are subject to problems of bacterial adhesion and subsequent biofilm formation, also known as implant-related infection, which leads to implant failure^2^,^3^,^4^. The incidence of biomaterial-associated infection (BAI) in orthopaedics has been reported to be between 2% and 5% in recent years^5^. Accordingly, there is an urgent need to improve the surface performance of Ti-based implants to hinder the formation of pathogen biofilms and promote tissue bio-integration with tissue^6^,^7^.

In order to improve the antimicrobial and anti-biofilm properties of implants, numerous processing methods have been developed for the surface modification of Ti-based implants^8^,^9^,^10^,^11^,^12^. One of the most common treatments is to coat the surface with antibiotics, but this approach may cause the drug-resistance and the emergence of deadly superbugs, such as methicillin-resistant *Staphylococcus aureus* (MRSA)^13^,^14^,^15^. As an alternative, inorganic antibacterial elements, such as silver (Ag) and copper (Cu), have also been used to offset the above concerns, especially Ag, which is widely known to exhibit broad-spectrum bactericidal or bacteriostatic activity at low concentrations without producing resistant bacteria^16^,^17^,^18^,^19^. In our previous work, silver nanoparticles (Ag NPs) fixed on Ti-based biomaterials through plasma immersion ion implantation (PIII) showed excellent anti-biofilm activity against adhesive bacteria, but this contact-killing surface could not exert an antiseptic effect on planktonic bacteria due to a lack of silver ion release^8^. However, if a slow and persistent release of silver ions (Ag^+^) could be achieved through careful design of the Ag NPs based on existing methods, then we hypothesize that the resultant modified coating would provide long-term antibacterial efficacy, effectively killing planktonic bacteria and preventing the surface adhesion of pathogens simultaneously. At the same time, it must be considered that excessive levels of Ag^+^ have the potential to demonstrate cytotoxicity^20^,^21^,^22^; thus, the concentration of the released Ag^+^ should strike a balance between tissue biocompatibility and pathogen-killing efficacy^23^. At present, numerous methods are available for loading implant surfaces with Ag, such as physical vapor deposition (PVD), PIII, plasma electrolytic oxidation (PEO), and chemical reduction, etc. However, it may be not easy to control Ag loading capacity using PVD, and it is difficult to release Ag^+^ from Ag NPs deposited by PIII because they typically become embedded in the Ti surface. Compared with these physically processing methods, hydrothermal chemistry method (HCM) offers certain advantages, such as controllable loading capacity, adjustable surface morphology, etc.^24^,^25^,^26^. This inspired us to investigate the HCM as a promising strategy for loading Ag onto Ti surface. In addition, a PEO-fabricated titania (TiO_2_) coating was selected for the pre-modifying layer on the Ti surface because PEO can produce porous, homogeneous and firmly adherent titania layers with favorable biocompatibility and bioactivity^27^,^28^,^29^,^30^. Moreover, previous literatures have manifested that inorganic ions deposited on the porous titania coating could present a pattern of sustained release instead of explosive release^31^,^32^.

Previous studies of the anti-biofilm properties of modified biomaterials have concentrated on the *ica*-dependent biofilm mechanism which plays an undeniably important role in staphylococcal biofilm development^8^,^33^; however, increasing evidences begin to emphasize the existence of an *ica*-independent biofilm mechanism, which is also involved in the pathogenesis of biomaterial-related infections, in both *S. aureus* and *S. epidermidis*^34^,^35^. This novel biofilm phenotype, as recently identified in a research on MRSA strains, is facilitated by the fibronectin (Fn)-binding proteins (FnBPs), i.e., FnBPA and FnBPB^36^. Hence, we sought to investigate whether a well-decorated Ti surface could exert their resistant effects against both the *ica*-dependent and FnBP-promoted biofilm mechanisms.

A surgical procedure offers an opportunity for contaminating bacteria to enter the acute wound, and any inserted surgical devices are also subject to consequent bacterial colonization. Thus, the fate of an implant surface may be pictured as a race between peri-implant tissue cells and invading bacteria^37^. If the bacteria win, the implant will be occupied by a biofilm and have to be replaced, and host tissues around the implant will also be destroyed by virulent pathogens^38^,^39^. However, if host tissue cells can availably cover the surface before their opponents, this will make the implant less vulnerable to bacteria and serve as the effective safeguard against implant infection^39^,^40^. Considering the bacterially contaminated perioperative period^41^, we intended to discover whether our proposed modified coating could help mammalian cells to win the race for occupying the implant surface; and if so, the prophylaxis for BAI could be perfectly implemented.

To this end, we first prepared porous titania coatings on Ti surfaces, followed by hydrothermal treatment to refine titania grains and deposit Ag nanoparticles, with the intent of compensating for the potential cytotoxicity of Ag^+^ by means of a synergistic effect between the hierarchical topography and chemical composition of the surface. On this basis, we then investigated the prepared surfaces to systematically evaluate the antimicrobial activity, biofilm resistance, and related genes expression for two types of biofilms. Moreover, we determined whether the fibroblast cells were favoured with respect to occupation of the modified surfaces through an *in vitro* bacteria-cell co-culture experiment.

## Results

### Sample characterization

**Figure 1:**
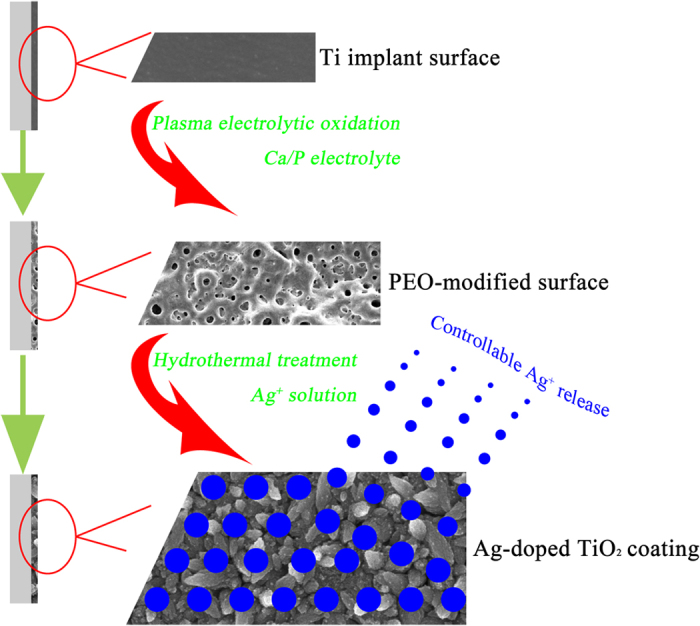
Schematic illustration of the fabrication process for the samples, including plasma electrolytic oxidation and hydrothermal chemical treatment.

**Figure 2:**
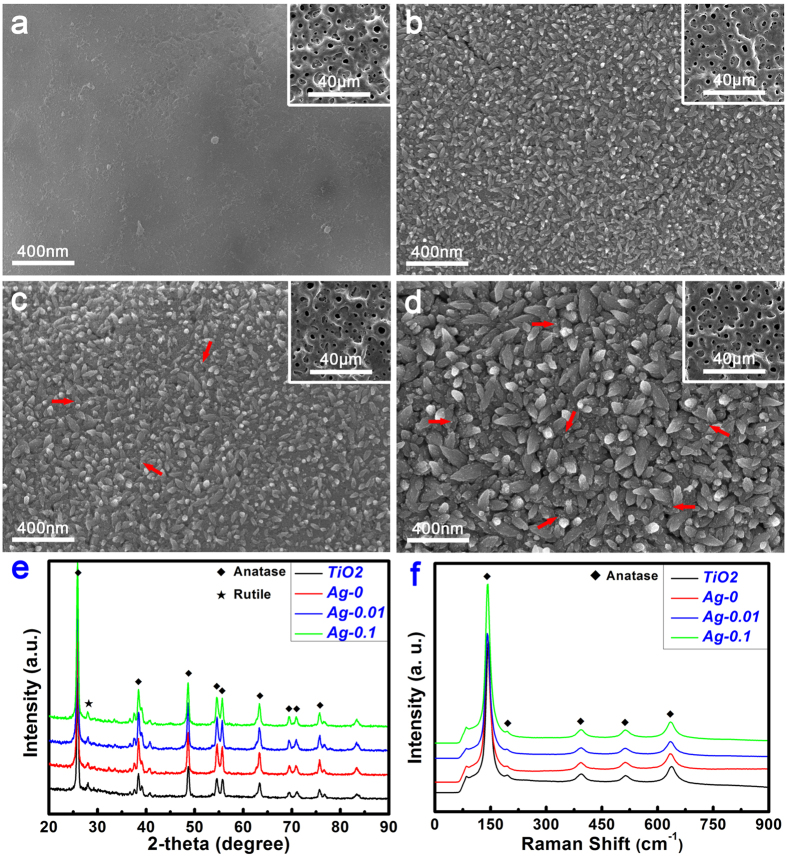
Surface morphologies of the TiO_2_ (**a**), Ag-0 (**b**), Ag-0.01 (**c**) and Ag-0.1 (**d**) samples and the corresponding XRD patterns (**e**) and Raman spectra (**f**) acquired from the surfaces of the various samples. The red arrows in (**c**) and (**d**) indicate Ag NPs.

The entire process of fabricating specimens is illustrated in Fig. 1; this process consisted of a two-step strategy of plasma electrolytic oxidation for the formation of TiO_2_ coatings and hydrothermal chemical treatment for the loading of Ag nanoparticles. As shown in Fig. 2a, for the TiO_2_ samples, a relatively smooth topography was observed on the Ti surface at high magnification after the PEO treatment. At low magnification (inset), the Ti surface exhibited a porous morphology, a typical characteristic of this surface modification method. By contrast, for the Ag-0 samples, a unique nanoawl structure, nearly perpendicular to Ti surface, was produced by the hydrothermal treatment in ultrapure water (Fig. 2b), although the porous topography was not obviously altered. It is inferred that the nanoawls originated from the refinement of the grains during the hydrothermal treatment. Furthermore, the grain size increased with the addition of silver acetate, as shown in Fig. 2c (Ag-0.01 sample). It can also be seen that tiny nanoparticles were deposited on the nanoawls. This could be observed more clearly for the Ag-0.1 samples (Fig. 2d). However, their low-magnification porous morphology was not significantly changed. According to the energy disperse spectroscopy (EDS) analysis results presented in Table 1, the Ag loading amounts for the Ag-0.01 and Ag-0.1 samples were 0.24 wt% and 2.29 wt%, respectively. As seen from the X-ray diffractometer (XRD) patterns in Fig. 2e, for all samples, the surface coatings predominantly consisted of anatase-phase titania and rutile-phase titania. Interestingly, according to the Raman spectra in Fig. 2f, the outermost surface layers of these samples contained only the anatase phase, particularly in the case of the hydrothermally treated samples, as the peaks at 145 cm^−1^, 197 cm^−1^, 399 cm^−1^, 518 cm^−1^ and 638 cm^−1^ are all associated with vibrations in anatase titania crystal^42^.

**Figure 3:**
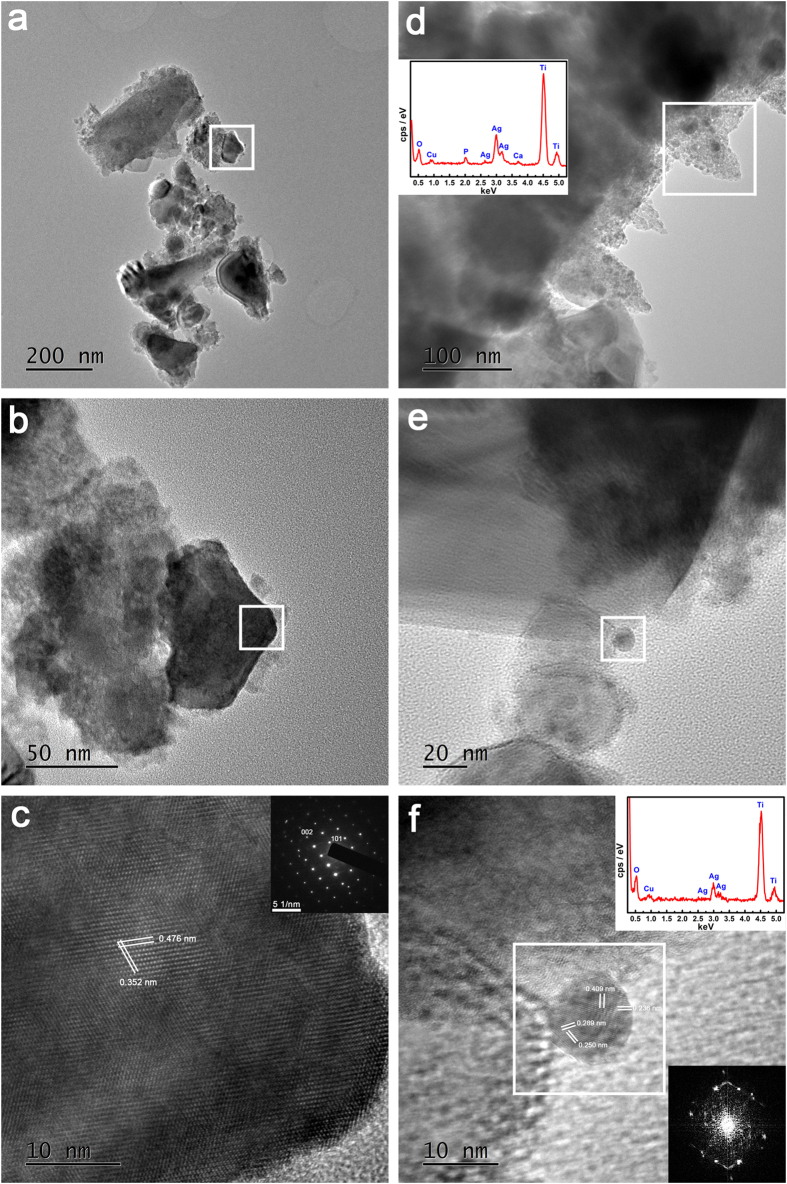
TEM analysis results for TiO_2_ (**a–c**) and Ag-0.1 (**d–f**) samples, including morphologies, HRTEM results, SAED results and the results of EDS elemental analysis.

To investigate the form in which the silver component was present on the surface, the transmission electron microscope (TEM) analysis was performed on the Ag-0 and Ag-0.1 samples. Figure 3a,b shows typical bright-field TEM images of the titania nanoawls scratched off from the surfaces of Ag-0 samples. The corresponding high-resolution image in Fig. 3c clearly reveals a set of fringes, indicating the anatase single-crystalline nature of the titania nanoawls, which is consistent with the selected area electron diffraction (SAED) pattern shown in the inset. With regard to the Ag-0.1 samples, as shown in Fig. 3d, bright-field imaging revealed the presence of tiny nanoparticles. Combined with the *in situ* EDS analysis results, the findings confirmed these tiny nanoparticles to be silver nanoparticles. Figure 3e provides a more detailed depiction of the bright-field features of the tiny nanoparticles. Upon *in situ* EDS analysis, the tiny nanoparticles in Fig. 3f were verified to be silver nanoparticles. The high- resolution fringes and the corresponding SAED pattern further indicated the successful deposition of metallic silver nanoparticles on the titania nanoawls.

**Figure 4:**
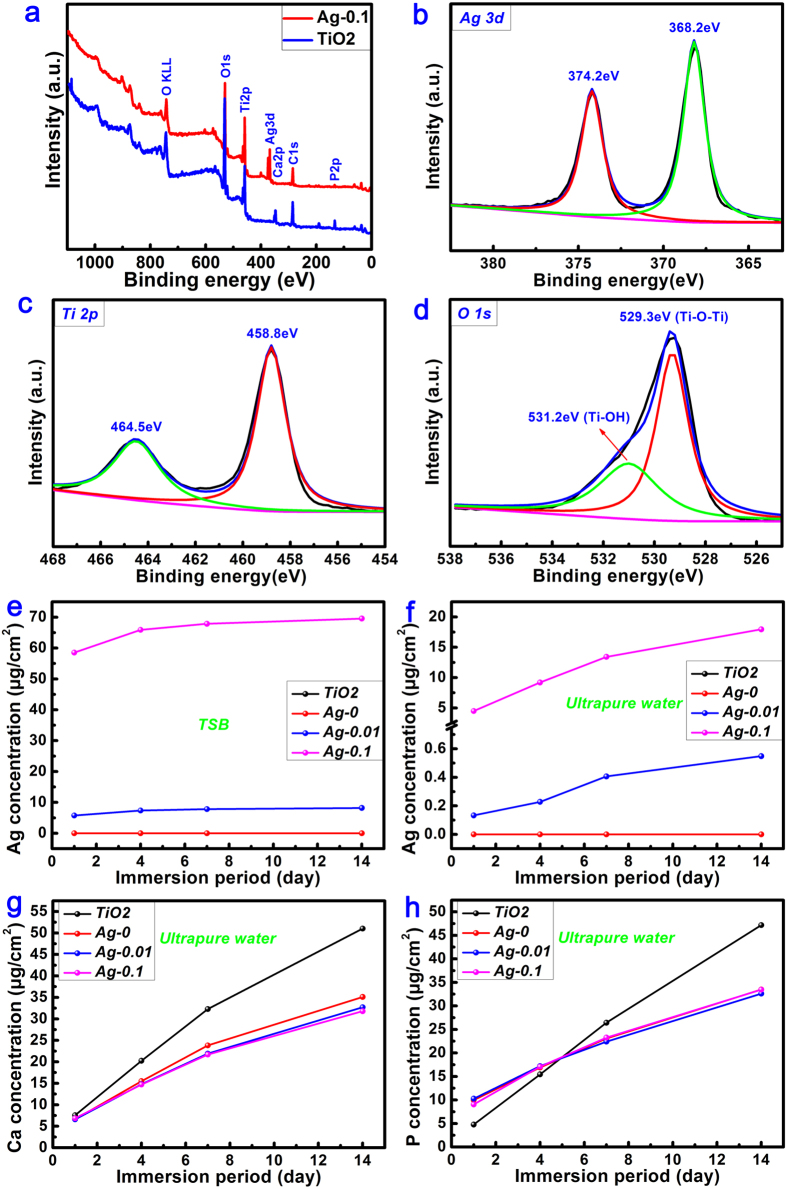
Full XPS spectra (**a**) acquired from the surfaces of TiO_2_ and Ag-0.1 samples, accompanied by the corresponding high-resolution Ag 3d (**b**), Ti 2p (**c**) and O 1s (**d**) XPS spectra. Release profiles of free silver matter in TSB (**e**) and ultrapure water (**f**) and the concomitant release of calcium ions (**g**) and phosphorus ions (**h**) in ultrapure water over 14 days.

Taking the Ag-0.1 samples as an example, the X-ray photoelectron spectroscopy (XPS) analysis was also conducted to further investigate the surface chemical composition and valence state. As shown in Fig. 4a, elements of Ti, oxygen (O), calcium (Ca) and phosphorus (P) were detected in the TiO_2_ coating, and after silver deposition via the hydrothermal method, the presence of silver could be clearly observed from the full XPS spectrum. Figure 4b shows the high-resolution Ag 3d XPS spectrum. The Ag 3d doublets centred at 368.2 eV for Ag 3d_5/2_ and 374.2 eV for Ag 3d_3/2_, with a spin energy separation of 6.0 eV, were assigned to metallic silver^43^,^44^, consistent with the TEM analysis (Fig. 3f). The high-resolution Ti 2p XPS spectrum, as shown in Fig. 4c, also contained two peaks, at 464.5 eV for Ti 2p_1/2_ and 458.8 eV for Ti 2p_3/2_ in the titania^45^. Similarly, the O 1s XPS spectrum (Fig. 4d) could be divided into two peaks at 529.3 eV for Ti-O-Ti and 531.2 eV for Ti-OH^46^.

In addition, the ion release profiles for various elements, including silver, calcium, phosphorus and titanium were detected via inductively coupled plasma optical emission spectrometry (ICP-OES). As shown in Fig. 4e, both Ag-0.01 and Ag-0.1 samples were found to exhibit sustained release of free silver ions in agar-free trypticase soy broth (TSB) medium over the 14 d of immersion. An obvious gap between the Ag-0.01 and Ag-0.1 samples can also be seen in this figure, which was due to their different levels of loading with free silver matter. This was also true when ultrapure water was used as the incubation medium (Fig. 4f). In addition, as seen from Fig. 4g,h, both calcium and phosphorus ions were released from all sample surfaces during the investigated period; however, no titanium species were detected.

### *In vitro* bioactivity

**Figure 5:**
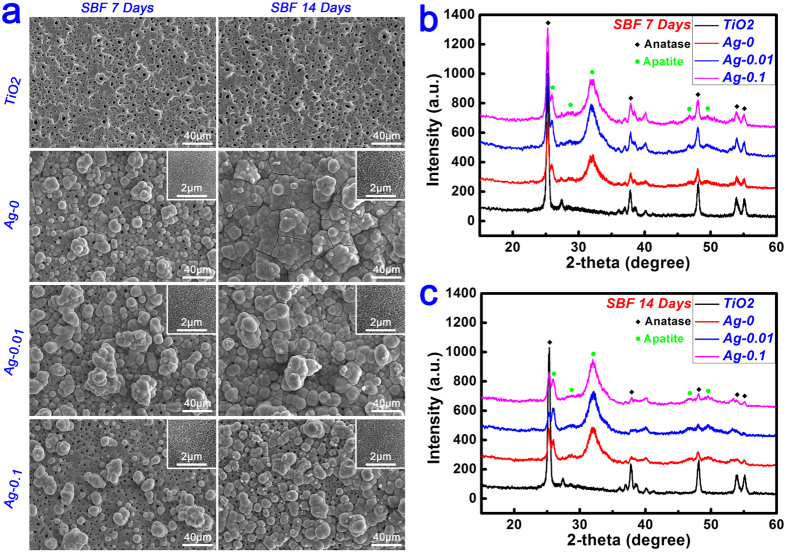
(**a**) Evolution of the surface morphologies of TiO_2_, Ag-0, Ag-0.01 and Ag-0.1 samples after immersion in SBF for 7 days and 14 days. (**b–c**) Surface XRD patterns of TiO_2_, Ag-0, Ag-0.01 and Ag-0.1 samples after immersion in SBF for 7 days and 14 days.

The apatite-forming ability of an implant in a physiological environment plays an important role in cell response and osseointegration. An *in vitro* immersion test in simulated body fluid (SBF) is typically conducted to estimate the surface bioactivity of a biomaterial. As shown in Fig. 5a, after 7 d of immersion in SBF, the TiO_2_ samples retained a bare surface morphology because of the bioinertia of the coating^1^. By contrast, spherical clusters appeared on the surfaces of the Ag-0 samples, consisting of subtle wormlike structures at high magnification (inset). As shown in Fig. 5b, characteristic diffraction peaks of crystalline apatite were clearly detected at 2θ = 26° and 32° in the XRD pattern, indicating that these sphere-like particles were assemblies of tiny apatite crystals. As for the Ag-0.01 samples, the surfaces were also partially covered with a discontinuous layer of spherical apatite clusters. However, the surface bioactivity of the Ag-0.1 samples was decreased compared with the Ag-0.01 samples. This was further confirmed by the relative changes in the intensities of the peaks corresponding to apatite (at 2θ = 32°) and anatase (at 2θ = 25.2°) in Fig. 5b. With the prolongation of the immersion time to 14 d, the TiO_2_ samples still remained bare, without any apatite clusters on their surface. On the contrary, the surfaces of both the Ag-0 and Ag-0.01 samples were completely covered with a newly formed layer of spherical apatite clusters exhibiting subtle a wormlike texture at high magnification, demonstrating their good bioactivity. In comparison, the surfaces of the Ag-0.1 samples were only partially covered with a discontinuous apatite layer. As expected, these observations were consistent with the results for 7 d of immersion, as further supported by the changes in the relative intensities of the anatase peak at 2θ = 25.2° and the apatite peak at 2θ = 32° in Fig. 5c.

### Antimicrobial and anti-biofilm properties

In this test, *S. epidermidis* and *S. aureus*, which are the most common microorganisms involved in orthopaedic implant infection and biofilm formation, were utilized to investigate the antimicrobial and anti-biofilm properties of our samples^35^,^47^. *S. epidermidis* (RP62A) and *S. aureus* (USA300) were selected as representatives of the two types of biofilm formation mechanisms, i.e., *ica*-dependent and *ica*-independent mechanisms, respectively^8^,^48^.

### Biocidal performance against planktonic bacteria

**Figure 6:**
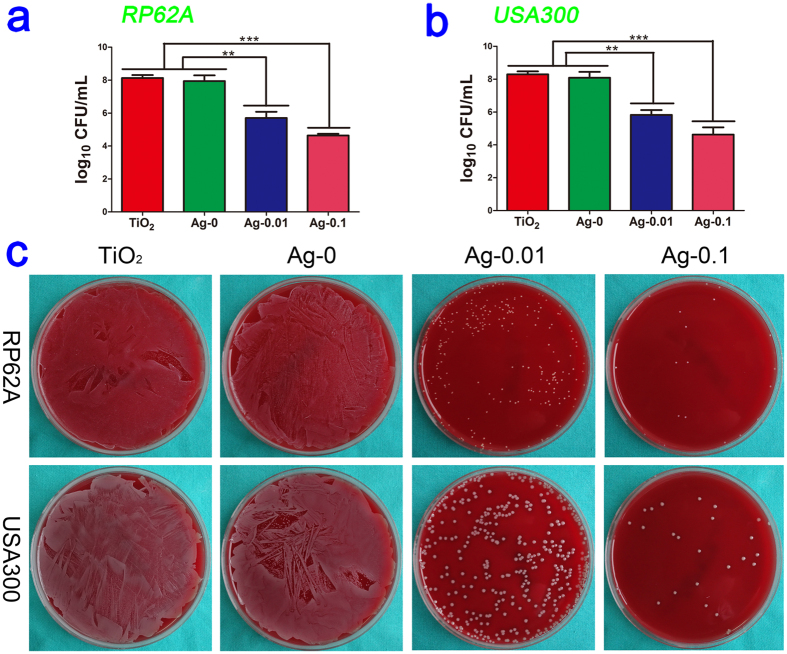
Antibacterial properties against planktonic bacteria *in vitro* at the end of the experiment. Subfigures (**a**) and (**b**) present the quantitative antibacterial effects on the CFU values in the media for RP62A and USA300, respectively. Significantly decreased CFU/ml counts of floating bacteria were observed. **P < 0.01, ***P < 0.001. Subfigure (**c**) shows typical photographs of two types of re-cultivated planktonic bacteria colonies on SBA plates.

As shown in Fig. 6a,b, after 18 h of exposure, the amounts of the planktonic bacteria *S.epidermidis* (RP62A, CFU/ml) decreased from 8.1 in the TiO_2_ control and 8.0 in the Ag-0 group to 5.7 in the Ag-0.01 group (*P* < 0.01) and 4.6 in isolates exposed to Ag-0.1 (*P* < 0.001). Moreover, the amounts of *S.aureus* (USA300, CFU/ml) decreased from 8.3 in the control and 8.2 in the Ag-0 specimens to 5.8 and 4.6 in the silver-exposed isolates (Ag-0.01, *P* < 0.01, and Ag-0.1, *P* < 0.001, respectively). These results indicate that TiO_2_-Ag (Ag-0.01 and Ag-0.1) samples exert damaging effects on the planktonic bacteria. Moreover, compared with the Ag-0.01 group, the Ag-0.1 group appeared to present slightly stronger antibacterial activity, although there was no statistically significant difference between them. The antibacterial efficiency against swimming bacteria was also qualitatively evaluated based on the representative spread plate method (SPM) pictures. As shown in Fig. 6c, the numbers of live bacteria in the TiO_2_ and Ag-0 groups were far greater than those in the TiO_2_-Ag groups, and the trend is consistent with the above quantitative results described above.

### Antimicrobial activity against sessile bacteria

**Figure 7:**
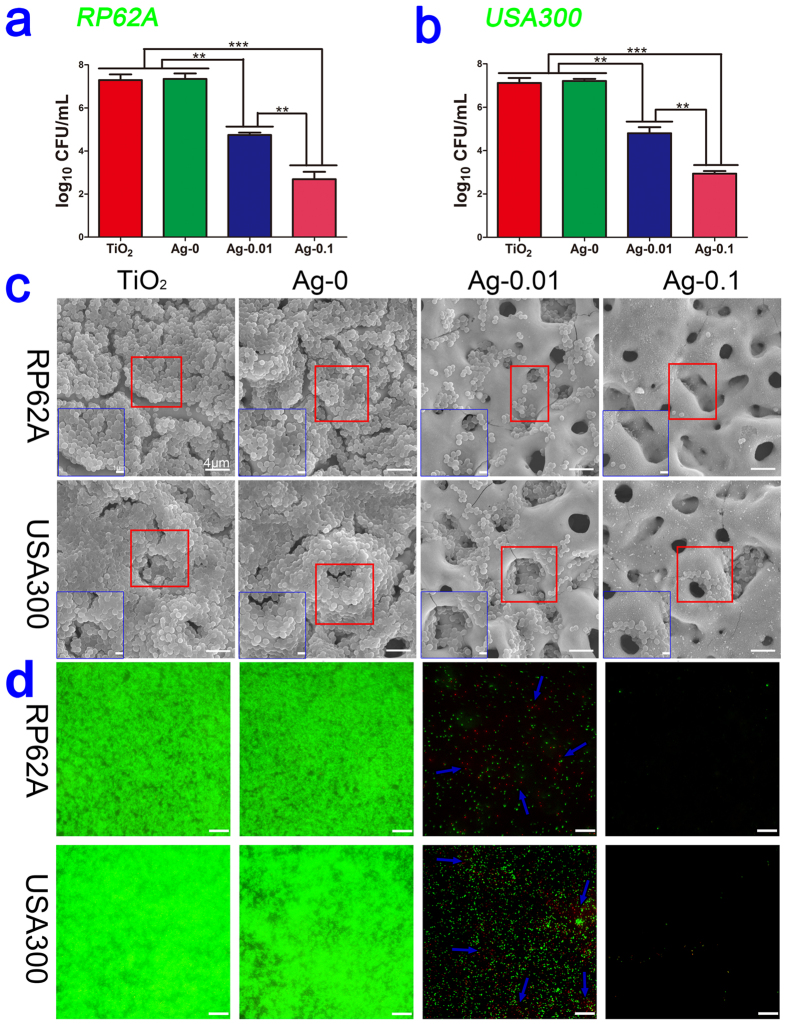
Antibacterial capability against sessile bacteria *in vitro* at the end of the experiment. Subfigures (**a**) and (**b**) present the quantitative antibacterial effects on the CFU value on the samples for RP62A and USA300, respectively. Significantly decreased CFU/ml counts of adherent bacteria were observed. **P < 0.01, ***P < 0.001. Subfigure (**c**) shows the SEM morphologies of the two types of biofilms on four specimen surfaces. The inner blue box corresponds to ×10000 magnification. The outer large box corresponds to ×3000 magnification. Subfigure (**d**) shows projected top views of the two types of biofilms on four sample surfaces obtained via CLSM at ×400 magnification. The scale bar represents 25 μm. The blue arrows indicate red fluorescence in the images.

As infecting organisms, *S. epidermidis* and *S. aureus* are usually either introduced during the insertion of implants or derived from a transient bacteraemia; and they can then adhere to the surface of implants and form a biofilm, namely, an organized bacterial consortium enclosed in a self-produced extracellular matrix^35^. Hence, the bacterial biofilm plays a very crucial role in the pathogenesis of implant-related infection. In our experiment, as shown in Fig. 7a,b, we found that after culturing overnight, the amounts of *S.epidermidis* (RP62A, CFU/ml) decreased from 7.3 in both the TiO_2_ control and Ag-0 groups to 4.7 in the Ag-0.01 samples (*P* < 0.01) and 2.7 in the Ag-0.1 samples (*P* < 0.001). And the amounts of *S.aureus* (USA300, CFU/ml) decreased from 7.1 in the control group and 7.2 in the Ag-0 group to 4.8 and 2.9 for the silver-containing specimens (Ag-0.01, *P* < 0.01,and Ag-0.1, *P* < 0.001, respectively). Compared to the Ag-0.01 samples, the Ag-0.1 samples presented stronger antibacterial activity (*P* < 0.01). This suggests that TiO_2_ -Ag samples are effective in preventing bacterial colonization on their surfaces. This trend of inhibiting bacterial adhesion was also verified by scanning electron microscopy (SEM) and confocal laser scanning microscopy (CLSM) observations for both kinds of bacteria. As observed from the SEM profiles, as shown in Fig. 7c, many bacteria adhered to the surfaces of the TiO_2_ and Ag-0 samples, aggregating into grapelike colonies to develop a biofilm. In contrast, not many bacteria were present on the surfaces of the Ag-0.01 samples, indicating that these specimens could prevent bacteria adherence and biofilm formation. Moreover, almost no bacterial cells could be observed on the Ag-0.1 samples, indicating no biofilm formation. In the CLSM images presented in Fig. 7d, we observed intense green fluorescence but only feeble red fluorescence in the TiO_2_ and Ag-0 samples, which indicated the presence of a sea of live bacteria attached to the surfaces and distinct biofilm formation, whereas, moderate green fluorescence and slightly more red fluorescence could be observed on the surfaces of the Ag-0.01 specimens, and relatively little green and red fluorescence could be detected on the Ag-0.1 surfaces. These findings implied less bacterial adhesion and a lower level of biofilm development on the Ag-0.01 surfaces and the adhesion of very few bacteria and nearly no biofilm formation on the Ag-0.1 surfaces. These observations were consistent with the results acquired by SEM.

### Analyses of the expression of biofilm-related genes

**Figure 8:**
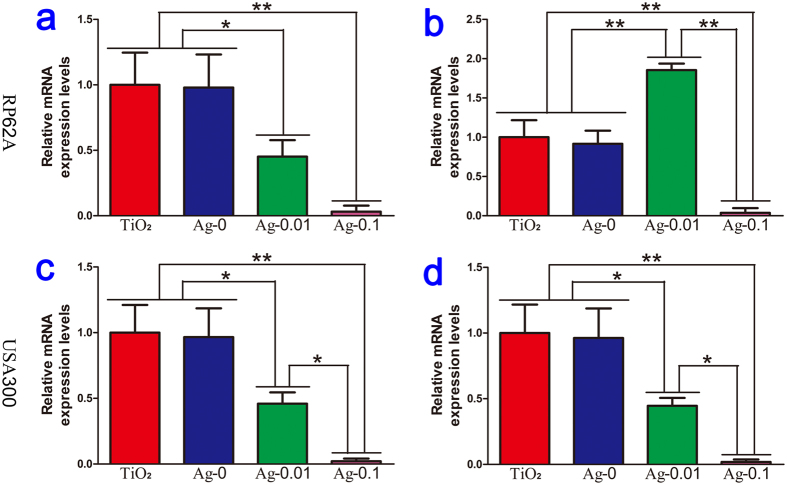
Relative levels of *ica*A (**a**) and *ica*R (**b**) expressed by *S. epidermidis* and *fnb*A (**c**) and *fnb*B (**d**) expressed by *S. aureus* cultured with four different samples. The expression levels of the biofilm genes are normalized with respect to the 16S rRNA gene. *P < 0.05, **P < 0.01.

Quantitative real-time PCR analysis was implemented to investigate whether our specimens could exert an effect on the expression of key genes during biofilm formation. Here, 16s rRNA was used for normalization, and the outcomes were formulated as gene expression levels relative to the TiO_2_ group. The expression levels of *ica*A and *ica*R, which participate in RP62A biofilm formation, are shown in Fig. 8a,b. Compared with those in the TiO_2_ and Ag-0 groups, the expression level of *ica*A was dramatically decreased in the Ag-0.01 group (*P* < 0.05), but the expression of *ica*R was markedly upregulated in the Ag-0.01 group (*P* < 0.01). Moreover, regarding USA300 biofilm formation, *fnb*A and *fnb*B gene expression analyses indicated that the Ag-0.01 samples could suppress biofilm formation by downregulating the expression of *fnb*A and *fnb*B, as shown in Fig. 8c,d. Interestingly, during both types of biofilm formation, the expression levels of all involved genes in the Ag-0.1 group were close to 0, which indicated that the inoculated bacteria were exposed to sufficient silver during the early stage that the growth of the bacteria was inhibited and their RNA could not be harvested at the end of incubation.

### *In vitro* cytocompatibility

**Figure 9:**
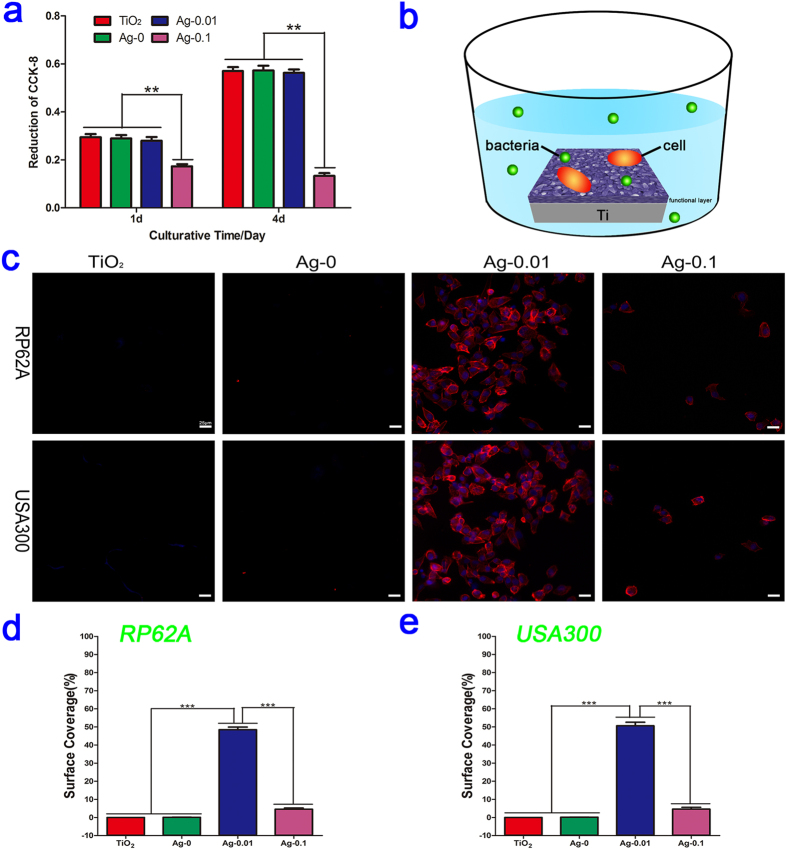
Cytotoxicity test and antibacterial assessment in a bacteria-fibroblast co-culture system. (**a**) Results of a CCK-8 assay showing the cytocompatibility for HT1080 cells cultured on micro-arc-oxidized TiO_2_ coatings with and without Ag doping. **P <  0.01 vs the Ag-0.1 group. (**b**) Schematic illustration of the co-culturing process for the fibroblasts, bacteria and samples. (**c**) Fluorescent images of fibroblast cells on four different specimens contaminated with PR62A or USA300 after staining with DAPI (blue) and TRITC-phalloidin (red). (**d–e**) The corresponding surface coverages of the four samples contaminated with RP62A or USA300. ***P < 0.001.

HT1080 cells were employed to evaluate the cytotoxicity of various specimens by means of the Cell Counting Kit-8 (CCK-8) assay. As shown in Fig. 9a, no statistically significant difference relative to TiO_2_ (control) was found for the Ag-0 and Ag-0.01 groups at each time point, which indicated that the titania micropore/nanoawl coatings and the subsequently formed Ag-doped(0.01 g/l) titania coating exhibited no obvious cytotoxicity and thus can be considered suitable for the potential applications. However, in the course of the experiment, the Ag-0.1 group exerted an obvious cytotoxic effect on the fibroblast cells, suggesting that excessive Ag content can still induce cytotoxicity; thus the Ag-0.1 specimens were deemed unfit for further study, although the Ag-0.1 specimens displayed the strongest antibacterial and anti-biofilm capability.

### *In vitro* bacteria-fibroblasts co-culture study

In comparison with previous mono-culture studies using only with either mammalian cells or bacteria, our bacteria-fibroblast co-culture study, as illustrated in Fig. 9b, was better representative of the conditions of the clinical practice. The adhesion and growth of the fibroblasts were analysed based on immunocyto-stained fluorescent images (ISFIs). After 36 h, as shown in Fig. 9c, the TiO_2_ and Ag-0 surfaces showed no living fibroblast cells. Few cells, with a wizened morphology, were present on the Ag-0.1 surfaces. However, a large number of robust fibroblasts attached to the surfaces of the Ag-0.01 samples. As depicted in Fig. 9d,e, in terms of the surface coverage by the cells, there was no fibroblast surface coverage on the TiO_2_ or Ag-0 surfaces, demonstrating that the presence of adherent bacteria affected the survival of fibroblast cells. The percentages of surface coverage on the Ag-0.1 samples were only 4.6% and 4.6% for RP62A and USA300, respectively. However, for the RP62A- and USA300- contaminated surfaces, the surface coverage percentages on the Ag-0.01 samples reached 48.5% and 50.6%, respectively.

## Discussion

As shown in Fig. 2, obvious titania nanoawl structures were produced on the surfaces of the coatings by the hydrothermal treatment. Meanwhile, Ti-OH groups were also formed on the surfaces during this process. Both the titania nanoawls and the Ti-OH groups synergistically contributed to the surface bioactivity to induce apatite formation^26^. During immersion in SBF, Ti-OH interacts with OH^−^ in the SBF to produce a negatively charged surface with Ti-O^−^ terminal groups (Ti − OH + OH^−^ → Ti − O^−^ + H_2_O), which are beneficial for apatite formation^49^. However, the deposition of silver nanoparticles can induce a positively shift in the surface charge thus degrading the surface bioactivity^8^, which may account for the decreased surface bioactivity of the Ag-0.1 samples.

When dissolved in bacterial solution, the Ag NPs deposited on the PEO-treated titania coatings could convert into Ag^+^, which could then kill microorganisms. Although the antibacterial mechanisms of Ag^+^ are not yet completely understood, it is generally recognized that Ag^+^ plays a critical role in binding with intracellular biological groups, such as proteins and nucleic acid, interfering with their function and ultimately leading to cell death^17^,^23^,^50^. In our tests of two Gram-positive bacteria, our TiO_2_-Ag samples demonstrated powerful biocidal activity against both, and with an increasing dose of silver, the antimicrobial efficacy became stronger.

Regarding the prominent anti-biofilm activity of the TiO_2_-Ag samples, we consider that there are two possible explanations, as follows. On the one hand, the independent effect of Ag NPs combined with their active physicochemical properties result in anti-biofilm activity^17^. Physical contact between Ag NPs and bacterial cell walls results in the formation of pits and is sufficient to produce a cytotoxic signal and induce cell death^51^,^52^. Moreover, direct contact of Ag NPs with bacterial cell membranes can damage the integrity of the membranes, causing the leakage of cellular components^53^. On the other hand, in addition to the antiseptic effect of the Ag NPs themselves, the Ag^+^ ions derived from the Ag NPs can attach to the bacterial cell walls and enter into cytoplasm to destroy the intracellular structures as a secondary effect^54^. Therefore, the investigated TiO_2_-Ag specimens can provide a systematic anti-biofilm capability for two typical kinds of bacteria. To further explore the specific anti-biofilm mechanisms, it is necessary to investigate the regulation effect of the modified surfaces on the expression levels of biofilm-related genes.

The complex mechanisms that drive the formation of a functional, mature staphylococcal biofilm are still poorly understood^35^. However, according to *in vitro* experimental models, it is generally recognized as a four-step process: (1) bacterial adhesion to the implant surface (2) bacterial aggregation into multiple cell layers (3) maturation of the biofilm architecture and (4) separation of cells from the mature biofilm into the floating state to start a new cycle of biofilm formation^35^,^55^. And at present, the prevention of biofilm formation can be achieved through two main approaches, namely, improved biomaterial coatings containing antimicrobial substances and modified implant surfaces with anti-adhesive properties^56^,^57^,^58^,^59^. Furthermore, the production of polysaccharide intercellular adhesin (PIA), whose synthesis is mediated by the intercellular adhesion (*ica*ADBC) locus, is thought to play an important role during the accumulative phase of the formation of a staphylococcal biofilm, and because the *ica* locus was initially discovered in *S. epidermidis,* for a long time it was even considered to be a primary virulence factor with regard to *S. epidermidis*-related implant infections^35^,^60^. However, differences in the biofilm formation mechanisms of *S. aureus* and *S. epidermidis* have been recognized^34^, and whereas carriage of the *ica* locus is strongly related to the biofilm-forming ability in *S. epidermidis* strains^60^, the relationship between *ica* locus carriage and the biofilm-forming ability in *S. aureus* is ambiguous, even though this locus also resides in almost all *S. aureus* strains^61^. Moreover, *ica*ADBC-independent biofilm mechanisms involving cell surface components have been described in *S. aureus*^62^ and a novel *S. aureus* biofilm phenotype mediated by the LPXTG-anchored FnBPs (where LPXTG is a specific recognition motif) has received increasing attention in recent researches, which indicate that the FnBP-promoted biofilm mechanism may be a pivotal determinant of virulence in implant infection^36^,^63^,^64^.

In this work, our experimental data demonstrated that Ag-incorporated titania coatings can effectively inhibit two types of biofilm formation by regulating the expression levels of related genes. For *S. epidermidis* biofilms, since the co-expression of *ica*A and *ica*D is essential for full PIA synthesis^65^,^66^ and the *ica*A*DBC* operon is adversely regulated by the repressor encoded by an upstream gene *icaR*^67^,^68^, we examined *ica*A and *ica*R transcription levels as an index for the evaluation of biofilm formation. In our study, our real-time PCR results demonstrated that Ag-0.01 surfaces can downregulate the expression of *ica*A while upregulating the transcription level of *ica*R, and we could not detect the expression of *ica*A or *ica*R on the Ag-0.1 surfaces because of the low level of bacterial RNA present. For *S. aureus* biofilms, the *fnb*A and *fnb*B genes are required for FnBP-mediated biofilm development^36^; therefore, we assessed the expression levels of these genes. Our results showed that the Ag-0.01 samples significantly downregulated *fnb*A and *fnb*B expression. Therefore, we conclude that the inhibition of the transcription of biofilm- related genes gave rise to a reduction in PIA/FnBP production and thus decreased biofilm formation and enhanced the susceptibility of the sessile bacteria to the Ag.

By virtue of the simplicity and flexibility with which surface morphology and chemical composition can be adjusted, hydrothermal amelioration is regarded as a hopeful chemical processing method for endowing Ti-based biomaterials with increased functionality^25^. Moreover, the synergetic effect of the hierarchical surface construction of a titania micropore/nanoawl coating and the incorporation of the nutrient elements of calcium and phosphorus may offset the cytotoxicity of Ag through optimization of the material design and the Ag doping content. With regard to our novel bacteria-cell co-culture investigation, the obtained results demonstrate that once sufficient bacteria have invaded during the perioperative period, mammalian cells will not win the race for occupation of the implant surface without the action of antibacterial agents that can restrain biofilm formation, thus preventing implant infection. However, if the dose of antibacterial agents is too high, it may damage the cells themselves in addition to effectively killing bacteria. Our bacteria-fibroblast co-culture study demonstrated that our Ag-0.01 samples could help fibroblast cells to triumph over the two tested types of bacteria, striking a balance between anti-infective activity and cytocompatibility, which holds great promise for orthopaedic clinical applications.

## Materials and Methods

### Fabrication of specimens

Commercial pure Ti (Grade 1, purity>99.85 wt%) plates with dimensions of 10 × 10 × 1 mm^3^ and 20 × 20 × 1 mm^3^ were first ultrasonically cleaned, followed by PEO treatment to form a layer of titania coating on the Ti surface in an electrolyte containing calcium acetate monohydrate and glycerophosphate disodium salt pentahydrate. Subsequently, a hydrothermal treatment in silver acetate solution (0 g/L, 0.01 g/L or 0.1 g/L) was applied at 140 °C for 24 h in a Teflon-lined steel reaction vessel to deposit Ag nanoparticles. The PEO-derived titania coating was used as the control (designated as “TiO_2_”), and the hydrothermally treated samples described above were designated as “Ag-0”, “Ag-0.01”, and “Ag-0.1”, respectively.

### Surface characterization of specimens

The surface morphology of the specimens was examined via field emission scanning electron microscopy (FESEM; S-4800, HITACHI, Japan). Their crystallinity was investigated using an X-ray diffractometer (XRD; D/Max, Rigaku, Tokyo, Japan) fitted with a Cu Kα (λ = 1.541 Å) source at 40 kV and 100 mA, in the range of 2θ = 10~90° at a step size of 0.02°. Phase identification was performed using the standard JCPDS database. In the X-ray diffraction measurements, the glancing angle of the incident beam against the sample surface was fixed at 1°. Raman spectra were recorded from 0 to 1000 cm^−1^ using a Raman microscope system (LabRAM, Horiba Jobin Yvon, France) with an Ar-ion laser at 20 mW (514.5 nm) for excitation. The surface chemical composition and chemical state of were analyzed by X-ray photoelectron spectroscopy (XPS; PHI 5802, Physical Electronics Inc, Eden Prairie, MN) with an Mg Kα (1253.6 eV) source, and TEM analysis was performed using a field emission transmission electron microscope (TEM; JEM-2100F, JEOL Ltd, Tokyo, Japan) with an accelerating voltage of 200 kV. Each sample for examination was scratched off from the surface of a specimen and dispersed in ethanol. A droplet of the resulting suspension was placed on a holey copper grid covered with a porous carbon film.

### Ion release measurement

The TiO_2_, Ag-0, Ag-0.01 and Ag-0.1 samples were soaked in 10 ml of agar-free TSB medium and fresh water in 15 ml sterile microcentrifuge tubes, followed by successive incubation for 1, 4, 7 and 14 d at 37 °C. After each incubation period, the leachates were collected and the amounts of silver, calcium, phosphorus and titanium ions released were determined via inductively coupled plasma optical emission spectrometry (ICP-OES, Varian Liberty 150, US).

### SBF test of apatite-forming ability

The TiO_2_, Ag-0, Ag-0.01 and Ag-0.1 samples were soaked in simulated body fluid (SBF) for 7 and 14 d to estimate their apatite-forming ability. The SBF was prepared by dissolving reagent-grade NaCl, NaHCO_3_, KCl, K_2_HPO_4_·3H_2_O, MgCl_2_·6H_2_O, CaCl_2_, and Na_2_SO_4_ in deionized water and was buffered to pH 7.4 with tris-hydroxymethyl-aminomethane (Tris) and HCl at 36.5 °C^26^. Two samples per group were soaked in a plastic vial containing 20 ml of SBF, and then placed in a biological thermostat at 36.5 °C under static conditions. At the end of the desired time, the samples were extracted from the SBF, gently washed with deionized water and allowed to dry naturally at room temperature.

### *In vitro* cytotoxicity evaluation

Fibroblast-like cells (HT1080, Shanghai Institute of Biological Science, Chinese Academy of Sciences) were utilized to determine the cytocompatibility of the samples and the cytotoxicity was analysed using the Cell Counting Kit-8 (CCK-8, Beyotime Bio-Tech, China) assay. The cells were grown in Dulbecco’s modified Eagle medium (DMEM, Gibco, USA) supplemented with 10% foetal bovine serum (FBS, Gibco, USA) and were maintained in a moist environment at 37 °C with 5% CO_2_. Each sample was placed in one well of a 24-well plate and cells with a density of 3 × 10^4^ cells/ml were seeded onto each sterile specimen. After 1 and 4 days of incubation, the culture medium was removed from the 24-well plate and each well was supplemented with 1 ml of fresh cell growth medium containing 100 μl of CCK-8 and placed in the incubator for 2 h. Finally, 100 μl of culture medium was transferred to a new 96-well plate, and the optical density was measured by a microplate reader (BIO-TEK, ELX 800) at 450 nm wavelength. All analyses were performed in triplicate.

### Bacterial source and culture

*S. epidermidis* (RP62A) was purchased in freeze-dried form from the American Type Culture Collection (Rockefeller, MD), and methicillin-resistant *S. aureus* (USA 300) was kindly provided by Dr. Binh An Diep (Department of Medicine, University of California, San Francisco, USA). For our test, the two types of bacteria were grown in a trypticase soy broth (TSB; BD Biosciences, Franklin Lakes, NJ) medium at 37 °C overnight. Then, the concentration of the *S. epidermidis* suspension was adjusted to ~1 × 10^6^ colony forming units (CFU)/ml based on the McFarland standard in TSB containing 1% (w/v) glucose (TSBG), and the concentration of the *S. aureus* suspension was also diluted to ~1 × 10^6^ CFU/ml based on the McFarland standard in 1%TSBG supplemented with human plasma. Human plasma was obtained from healthy adult donors at Shanghai Jiao Tong University Affiliated Sixth People’s Hospital. Our study protocol was approved by the Shanghai Jiao Tong University Affiliated Sixth People’s Hospital Ethics Committee, and it complies with the moral principles of the Helsinki declaration of 2000. All donors signed a written informed consent form before entry into our study. Sterile specimens were placed in new 24-well plates, and 500 μl of bacterial suspension was added into each well containing a specimen. Afterwards, the samples were statically incubated overnight (for 18 hours) at 37 °C for a subsequent evaluation of antibacterial activity.

### Quantitative and qualitative evaluation of antibacterial efficiency

At the end of our test, the amounts of viable planktonic bacteria present in original medium were examined through the SPM (spread plate method, namely, bacterial suspensions were serially diluted with fresh PBS, evenly spread on sheep blood agar (SBA) plates, and incubated overnight at 37 °C.)^8^. The number of CFU on each SBA plate was calculated using the protocol specified by the Chinese National Standard GB/T 4789.2, and typical experimental results were photographically recorded for qualitative evaluation of the planktonic bacteria. Then, the specimens were extracted from the medium using sterile forceps, and any non-adherent bacteria on the samples were gently rinsed away with fresh PBS. Moreover, each foil sample was placed in 1 ml of fresh PBS and subjected to ultrasonic vibration (150 W, B3500S-MT, Branson Ultrasonics Co., Shanghai, China) for 5 min to separate the attached bacteria, and then, after vortex mixing, we drew a sample of the final suspension to calculate the live bacteria using the SPM. Each experiment was repeated three times.

### Qualitative evaluation of bacterial biofilms by SEM

The incubated samples were lightly washed twice with sterile PBS, fixed with 2.5% glutaraldehyde at 4 °C for 18 h, and sequentially dehydrated in the scalar ethanol series (50, 70, 80, 90,95 v/v%) once (for 15 min) and then in absolute ethanol twice (for 15 min each time). Then, the specimens were desiccated, sprayed with gold, and ultimately observed via scanning electron microscopy (SEM; JEOL JSM-6310LV, Japan).

### Qualitative evaluation of bacterial biofilms by CLSM

The incubated samples were lightly washed twice with sterile PBS, placed in a new 24-well plate, stained with 1 ml of mixed dye (LIVE/DEAD BacLight bacteria viability kits, Invitrogen) for 20 min, gently rinsed once with sterile PBS and finally imaged via confocal laser scanning microscopy (CLSM; LSM 510 meta, Zeiss, Germany). Live bacteria with intact cytomembranes were stained green, whereas dead bacteria with damaged cell membranes were stained red.

### qRT-PCR analysis of related gene transcription for two representative biofilms

The genes involved in the formation of the two types of biofilms were quantitatively tested via real-time polymerase chain reaction (PCR). We added 2 ml of bacterial suspension (10^6^ CFU/ml) into each well containing a sample (2 cm square) in a 6-well plate and statically cultivated the plate at 37 °C overnight. *S. epidermidis* RP62A was selected for the *ica*A and *ica*R expression assays and MRSA USA300 was selected for the *fnb*A and *fnb*B expression assays. After the incubation period, the adhering bacteria were collected and pelleted via centrifugation at 10,000 g for 5 min and then resuspended in 1 ml of PBS with 100 mg/ml of lysostaphin (Sigma) and incubated for 10 min at 37 °C^33^. Total RNA extraction was performed using the RNeasy Mini Kit (Qiagen, Germany). Then, 1 μg of the total RNA was reverse transcribed to its complementary DNA (cDNA) using the PrimeScript RT reagent kit (Takara), and qRT-PCR analysis was performed on a Bio-Rad C1000 system using SYBR Premix Ex Taq II (Takara). Commercially synthesized primers related to the target genes were used, as presented in Table 2. The expression levels of *ica*A*, ica*R, *fnb*A and *fnb*B were assessed and normalized with respect to that of the internal standard gene 16S rRNA. The quantification of the expression of the target genes was grounded on a cycle threshold value for each sample that was computed by averaging three replicate measurements^33^,^69^.

### *In vitro* co-culture experiments with bacteria and fibroblast cells

Based on previous research^7^,^40^, our *in vitro* co-culture study was performed by sequentially applying bacteria (RP62A/USA300) and fibroblast cells to the sample surfaces. First, 25 μl volumes of bacterial suspensions (10^5^ CFU/ml) were applied to the specimens and incubated for 90 min at 37 °C. Second, fibroblasts in modified culture medium were distributed on the sample surfaces at a density of 10^5^ cells/well in a 24-well plate and then incubated for 36 h in 5% CO_2_ at 37 °C. This modified medium contained cell growth medium and TSB. Ultimately, at the end of the experiment, the samples were fixed in 4% paraformaldehyde, stained with TRITC-phalloidin and DAPI, and imaged using a fluorescence microscope (Leica Microsystems LTD, Germany). The results were quantitatively evaluated based on the surface coverage of the foils by fibroblasts in the presence of contaminated bacteria, and the analysis was performed as described in previous reports.

### Statistical analysis

All studies were implemented in triplicate. All data are expressed as the mean ± standard deviation (SD). Under the assumption of normal distributions and equal variances, one-way ANOVA and SNK *post-hoc* analyses were applied to perform statistical comparisons between groups, and P values of less than 0.05 were considered statistically significant.

## Conclusion

In summary, TiO_2_ coating with a hierarchical hybrid micro/nanostructure of incorporated Ag-nanoparticles were first prepared on Ti surfaces to develop a delivery platform that could provide the sustained release of silver ions. Then, it was demonstrated that these modified surfaces exhibit good antimicrobial properties against both adherent bacteria and planktonic bacteria for two representative strains of epidemic *Staphylococcus* (*S. epidermidis*, RP62A; *MRSA*, USA 300). The modified surfaces can regulate the expression levels of biofilm-related genes (*ica*A and *ica*R for *S. epidermidis*; *fnb*A and *fnb*B for *MRSA*) to inhibit bacterial adhesion and biofilm formation. In addition, a bacteria-cell co-culture investigation revealed that the incorporation of Ag nanoparticles on a Ti surface can help mammalian cells to survive, adhere and spread more successfully than *Staphylococcus*, resulting in a mammalian cell-assisting antibacterial functionality. Besides, the modified surfaces also showed good bioactivity. This study represents a meaningful attempt to provide new insight into the antibacterial behavior and mechanisms of Ag-nanoparticle-functionalized Ti implant surfaces with bacteria-killing and cell-assisting functionalities, thereby paving the way towards better satisfying clinical needs in orthopedic applications.

## Additional Information

**How to cite this article**: Wang, J. X. *et al*. Silver-nanoparticles-modified biomaterial surface resistant to *staphylococcus*: new insight into the antimicrobial action of silver. *Sci. Rep.* **6**, 32699; doi: 10.1038/srep32699 (2016).

## Figures and Tables

**Table 1:** Elemental composition of various samples analyzed by EDS technique.

Sample	Elemental content (wt.%, Mean ± SD)				
	Ag	Ca	P	Ti	O
TiO_2_	—	7.62 ± 0.10	5.45 ± 0.13	47.43 ± 0.17	39.51 ± 0.22
Ag-0	0	2.94 ± 0.09	2.32 ± 0.13	58.64 ± 0.49	36.10 ± 0.40
Ag-0.01	0.24 ± 0.01	2.73 ± 0.11	2.26 ± 0.15	57.95±0.06	36.82 ± 0.07
Ag-0.1	2.29 ± 0.05	2.12±0.05	2.15 ± 0.03	59.15 ± 0.32	34.30 ± 0.35

**Table 2:** Primers used in the present study for real-time polymerase chain reaction.

Target gene	Direction	Primer sequence (5′→3′)
*ica*A	F	AACAAGTTGAAGGCATCTCC
*ica*A	R	GATGCTTGTTTGATTCCCT
*ica*R	F	CCATTGACGGACTTTACCAG
*ica*R	R	CAAAGCGATGTGCGTAGGA
*fnb*A	F	GAAGATACAAACCCAGGTGG
*fnb*A	R	GACCATTTTCAGTTCCTAAACCAG
*fnb*B	F	GAAGAAGATACAAACCCAGGTGG
*fnb*B	R	GTGACCATTTTCAGTTCCTAAACC
16S rRNA	F	TCGTGTCGTGAGATGTTGGGTTA
16S rRNA	R	GGTTTCGCTGCCCTTTGTATTGT

## References

[b1] Liu X., Chu P. K. & Ding C. Surface modification of titanium, titanium alloys, and related materials for biomedical applications. Mater. Sci. Eng. R. 47, 49–121 (2004).

[b2] Campoccia D., Montanaro L. & Arciola C. R. The significance of infection related to orthopedic devices and issues of antibiotic resistance. Biomaterial 27, 2331–2339 (2006). 10.1016/j.biomaterials.2005.11.044 16364434

[b3] Campoccia D., Montanaro L. & Arciola C. R. A review of the clinical implications of anti-infective biomaterials and infection-resistant surfaces. Biomaterials 34, 8018–8029 (2013). 23932292 10.1016/j.biomaterials.2013.07.048

[b4] Campoccia D., Montanaro L. & Arciola C. R. A review of the biomaterials technologies for infection-resistant surfaces. Biomaterials 34, 8533–8554 (2013). 23953781 10.1016/j.biomaterials.2013.07.089

[b5] Darouiche R. O. Treatment of infections associated with surgical implants. N Engl J Med. 350, 1422–1429 (2004). 15070792 10.1056/NEJMra035415

[b6] Huang R., Han Y. & Lu S. Enhanced osteoblast functions and bactericidal effect of Ca and Ag dual-ion implanted surface layers on nanograined titanium alloys. J. Mater. Chem. B. 2, 4531–4543 (2014). 10.1039/c4tb00124a 32261554

[b7] Yue C., Kuijer R., Kaper H. J., van der Mei H. C. & Busscher H. J. Simultaneous interaction of bacteria and tissue cells with photocatalytically activated, anodized titanium surfaces. Biomaterials 35, 2580–2587 (2014). 24393267 10.1016/j.biomaterials.2013.12.036

[b8] Qin H. . *In vitro* and *in vivo* anti-biofilm effects of silver nanoparticles immobilized on titanium. Biomaterials 35, 9114–9125 (2014). 25112937 10.1016/j.biomaterials.2014.07.040

[b9] Cao H. . Nano-thick calcium oxide armed titanium: boosts bone cells against methicillin-resistant Staphylococcus aureus. Sci.Rep. 6, 21761 (2016). 26899567 10.1038/srep21761 PMC4761977

[b10] Huang Y. . The construction of hierarchical structure on Ti substrate with superior osteogenic activity and intrinsic antibacterial capability. Sci. Rep. 4, 6172 (2014). 25146099 10.1038/srep06172 PMC4141259

[b11] de Avila E. D. . Effect of UV-photofunctionalization on oral bacterial attachment and biofilm formation to titanium implant material. Biomaterials 67, 84–92 (2015). 26210175 10.1016/j.biomaterials.2015.07.030 PMC4667792

[b12] Antoci V. Jr. . The inhibition of Staphylococcus epidermidis biofilm formation by vancomycin-modified titanium alloy and implications for the treatment of periprosthetic infection. Biomaterials 29, 4684–4690 (2008). 18814909 10.1016/j.biomaterials.2008.08.016 PMC2617720

[b13] Fujimura S. . Antimicrobial efficacy of combined clarithromycin plus daptomycin against biofilms-formed methicillin-resistant Staphylococcus aureus on titanium medical devices. J Infect Chemother. 21, 756–759 (2015). 26162777 10.1016/j.jiac.2015.06.001

[b14] Huh A. J. & Kwon Y. J. “Nanoantibiotics”: A new paradigm for treating infectious diseases using nanomaterials in the antibiotics resistant era. J Control Release 156, 128–145 (2011). 21763369 10.1016/j.jconrel.2011.07.002

[b15] Salwiczek M. . Emerging rules for effective antimicrobial coatings. Trends Biotechnol. 32, 82–90 (2014). 24176168 10.1016/j.tibtech.2013.09.008

[b16] Kim J. S. . Antimicrobial effects of silver nanoparticles. Nanomedicine 3, 95–101 (2007). 17379174 10.1016/j.nano.2006.12.001

[b17] You C. . The progress of silver nanoparticles in the antibacterial mechanism, clinical application and cytotoxicity. Mol Biol Rep. 39, 9193–9201 (2012). 22722996 10.1007/s11033-012-1792-8 PMC7089021

[b18] Chaloupka K., Malam Y. & Seifalian A. M. Nanosilver as a new generation of nanoproduct in biomedical applications. Trends Biotechnol. 28, 580–588 (2010). 20724010 10.1016/j.tibtech.2010.07.006

[b19] Swathy J. R. . Antimicrobial silver: An unprecedented anion effect. Sci. Rep. 4, 7161 (2014). 25418185 10.1038/srep07161 PMC4241523

[b20] de Lima R., Seabra A. B. & Duran N. Silver nanoparticles: a brief review of cytotoxicity and genotoxicity of chemically and biogenically synthesized nanoparticles. J Appl Toxicol. 32, 867–879 (2012). 22696476 10.1002/jat.2780

[b21] Albers C. E., Hofstetter W., Siebenrock K. A., Landmann R. & Klenke F. M. *In vitro* cytotoxicity of silver nanoparticles on osteoblasts and osteoclasts at antibacterial concentrations. Nanotoxicology 7, 30–36 (2013). 22013878 10.3109/17435390.2011.626538

[b22] Li J., Qiao Y., Zhu H., Meng F. & Liu X. Existence, release, and antibacterial actions of silver nanoparticles on Ag-PIII TiO_2_ films with different nanotopographies. Int J Nanomedicine 9, 3389–3402 (2014). 25075186 10.2147/IJN.S63807 PMC4106954

[b23] Chernousova S. & Epple M. Silver as antibacterial agent: ion, nanoparticle, and metal. Angew Chem Int Ed Engl. 52, 1636–1653 (2013). 23255416 10.1002/anie.201205923

[b24] Kulkarni M. . Titanium nanostructures for biomedical applications. Nanotechnology 26, 062002 (2015). 25611515 10.1088/0957-4484/26/6/062002

[b25] Li J. . Chemically regulated bioactive ion delivery platform on a titanium surface for sustained controlled release. J. Mater. Chem. B. 2, 283–294 (2014). 10.1039/c3tb21102a 32261507

[b26] Li J. . Enhanced bioactivity and bacteriostasis effect of TiO_2_ nanofilms with favorable biomimetic architectures on titanium surface. RSC Advances 3, 11214–11225 (2013).

[b27] Krzakala A., Kazek-Kesik A. & Simka W. Application of plasma electrolytic oxidation to bioactive surface formation on titanium and its alloys. RSC Advances 3, 19725–19743 (2013).

[b28] Han Y., Zhou J., Lu S. & Zhang L. Enhanced osteoblast functions of narrow interligand spaced Sr-HA nano-fibers/rods grown on microporous titania coatings. RSC Advances 3, 11169–11184 (2013).

[b29] Zhou J. H., Han Y. & Lu S. M. Direct role of interrod spacing in mediating cell adhesion on Sr-HA nanorod-patterned coatings. Int J Nanomedicine 9, 1243–1260 (2014). 24634585 10.2147/IJN.S58236 PMC3952902

[b30] Zhou J. & Han Y. Effect of hydrothermal treatment model on the formation of Sr-HA nanorod arrays on microarc oxidized titania coatings. Appl. Surf. Sci. 286, 384–390 (2013).

[b31] Lu P., Cao L., Liu Y., Xu X. & Wu X. Evaluation of magnesium ions release, biocorrosion, and hemocompatibility of MAO/PLLA-modified magnesium alloy WE42. J Biomed Mater Res B Appl Biomater 96, 101–109 (2011). 21053265 10.1002/jbm.b.31744

[b32] Jo J. H., Hong J. Y., Shin K. S., Kim H. E. & Koh Y. H. Enhancing biocompatibility and corrosion resistance of Mg implants via surface treatments. J Biomater Appl. 27, 469–476 (2012). 21862515 10.1177/0885328211412633

[b33] Tan H. . The use of quaternised chitosan-loaded PMMA to inhibit biofilm formation and downregulate the virulence-associated gene expression of antibiotic-resistant staphylococcus. Biomaterials 33, 365–377 (2012). 22014946 10.1016/j.biomaterials.2011.09.084

[b34] O’Gara J. P. ica and beyond: biofilm mechanisms and regulation in Staphylococcus epidermidis and Staphylococcus aureus. FEMS Microbiol Lett. 270, 179–188 (2007). 17419768 10.1111/j.1574-6968.2007.00688.x

[b35] Arciola C. R., Campoccia D., Speziale P., Montanaro L. & Costerton J. W. Biofilm formation in Staphylococcus implant infections. A review of molecular mechanisms and implications for biofilm-resistant materials. Biomaterials 33, 5967–5982 (2012). 22695065 10.1016/j.biomaterials.2012.05.031

[b36] O’Neill E. . A novel Staphylococcus aureus biofilm phenotype mediated by the fibronectin-binding proteins, FnBPA and FnBPB. J Bacteriol. 190, 3835–3850 (2008). 18375547 10.1128/JB.00167-08 PMC2395027

[b37] Gristina A. G. Biomaterial-centered infection: microbial adhesion versus tissue integration. Science 237, 1588–1595 (1987). 3629258 10.1126/science.3629258

[b38] Hall-Stoodley L., Costerton J. W. & Stoodley P. Bacterial biofilms: from the natural environment to infectious diseases. Nat Rev Microbiol. 2, 95–108 (2004). 15040259 10.1038/nrmicro821

[b39] Subbiahdoss G., Kuijer R., Grijpma D. W., van der Mei H. C. & Busscher H. J. Microbial biofilm growth vs. tissue integration: “the race for the surface” experimentally studied. Acta Biomater 5, 1399–1404 (2009). 19158003 10.1016/j.actbio.2008.12.011

[b40] Zhao B. . Soft tissue integration versus early biofilm formation on different dental implant materials. Dent mater 30, 716–727 (2014). 24793200 10.1016/j.dental.2014.04.001

[b41] Gurkan I. & Wenz J. F. Perioperative infection control: an update for patient safety in orthopedic surgery. Orthopedics 29, 329–339; quiz 340-321 (2006). 16628993 10.3928/01477447-20060401-13

[b42] Lottici P. P., Bersani D., Braghini M. & Montenero A. Raman scattering characterization of gel-derived titania glass. J Mater Sci. 28, 177–183 (1993).

[b43] Liu Y., Jordan R. G. & Qiu S. L. Electronic structures of ordered Ag-Mg alloys. Phys Rev B. 49, 4478–4484 (1994). 10.1103/physrevb.49.4478 10011367

[b44] Shalvoy R. B., Fisher G. B. & Stiles P. J. Bond ionicity and structural stability of some average-valence-five materials studied by x-ray photoemission. Phys Rev B. 15, 1680–1697 (1977).

[b45] Hu H. . Antibacterial activity and increased bone marrow stem cell functions of Zn-incorporated TiO_2_ coatings on titanium. Acta Biomater 8, 904–915 (2012). 22023752 10.1016/j.actbio.2011.09.031

[b46] Akhavan O., Azimirad R., Safa S. & Hasani E. CuO/Cu(OH)_2_ hierarchical nanostructures as bactericidal photocatalysts. J. Mater. Chem. 21, 9634–9640 (2011).

[b47] Shen H. . Pathogenic analysis in different types of orthopedic implant infections. Chin Med J (*Engl*). 127, 2748–2752 (2014). 25146607

[b48] Dastgheyb S., Parvizi J., Shapiro I. M., Hickok N. J. & Otto M. Effect of biofilms on recalcitrance of staphylococcal joint infection to antibiotic treatment. J Infect Dis. 211, 641–650 (2015). 25214518 10.1093/infdis/jiu514 PMC4318921

[b49] Liu X. . Plasma-treated nanostructured TiO_2_ surface supporting biomimetic growth of apatite. Biomaterials 26, 6143–6150 (2005). 15927251 10.1016/j.biomaterials.2005.04.035

[b50] Feng Q. L. . A mechanistic study of the antibacterial effect of silver ions on *Escherichia coli* and Staphylococcus aureus. J Biomed Mater Res. 52, 662–668 (2000). 11033548 10.1002/1097-4636(20001215)52:4<662::aid-jbm10>3.0.co;2-3

[b51] Choi O. . The inhibitory effects of silver nanoparticles, silver ions, and silver chloride colloids on microbial growth. Water Res. 42, 3066–3074 (2008). 18359055 10.1016/j.watres.2008.02.021

[b52] Choi O. K. & Hu Z. Q. Nitrification inhibition by silver nanoparticles. Water Sci Technol. 59, 1699–1702 (2009). 19448303 10.2166/wst.2009.205

[b53] Sondi I. & Salopek-Sondi B. Silver nanoparticles as antimicrobial agent: a case study on *E. coli* as a model for Gram-negative bacteria. J Colloid Interface Sci. 275, 177–182 (2004). 15158396 10.1016/j.jcis.2004.02.012

[b54] Volker C., Oetken M. & Oehlmann J. The biological effects and possible modes of action of nanosilver. Rev Environ Contam Toxicol. 223, 81–106 (2013). 23149813 10.1007/978-1-4614-5577-6_4

[b55] Costerton J. W., Montanaro L. & Arciola C. R. Biofilm in implant infections: its production and regulation. Int J Artif Organs 28, 1062–1068 (2005). 16353112 10.1177/039139880502801103

[b56] Lonn-Stensrud J., Landin M. A., Benneche T., Petersen F. C. & Scheie A. A. Furanones, potential agents for preventing Staphylococcus epidermidis biofilm infections? J Antimicrob Chemother . 63, 309–316 (2009). 19098295 10.1093/jac/dkn501

[b57] Shi Z. L., Chua P. H., Neoh K. G., Kang E. T. & Wang W. Bioactive titanium implant surfaces with bacterial inhibition and osteoblast function enhancement properties. Int J Artif Organs 31, 777–785 (2008). 18924089 10.1177/039139880803100905

[b58] Kazemzadeh-Narbat M. . Antimicrobial peptides on calcium phosphate-coated titanium for the prevention of implant-associated infections. Biomaterials 31, 9519–9526 (2010). 20970848 10.1016/j.biomaterials.2010.08.035

[b59] Zhang F., Zhang Z., Zhu X., Kang E. T. & Neoh K. G. Silk-functionalized titanium surfaces for enhancing osteoblast functions and reducing bacterial adhesion. Biomaterials 29, 4751–4759 (2008). 18829101 10.1016/j.biomaterials.2008.08.043

[b60] Ziebuhr W. . Detection of the intercellular adhesion gene cluster (ica) and phase variation in Staphylococcus epidermidis blood culture strains and mucosal isolates. Infect Immun. 65, 890–896 (1997). 9038293 10.1128/iai.65.3.890-896.1997 PMC175065

[b61] O’Neill E. . Association between methicillin susceptibility and biofilm regulation in Staphylococcus aureus isolates from device-related infections. J Clin Microbiol. 45, 1379–1388 (2007). 17329452 10.1128/JCM.02280-06 PMC1865887

[b62] Houston P., Rowe S. E., Pozzi C., Waters E. M. & O’Gara J. P. Essential role for the major autolysin in the fibronectin-binding protein-mediated Staphylococcus aureus biofilm phenotype. Infect Immun. 79, 1153–1165 (2011). 21189325 10.1128/IAI.00364-10 PMC3067512

[b63] O’Neill E., Humphreys H. & O’Gara J. P. Carriage of both the fnbA and fnbB genes and growth at 37 degrees C promote FnBP-mediated biofilm development in meticillin-resistant Staphylococcus aureus clinical isolates. J Med Microbiol. 58, 399–402 (2009). 19273632 10.1099/jmm.0.005504-0

[b64] Vergara-Irigaray M. . Relevant role of fibronectin-binding proteins in Staphylococcus aureus biofilm-associated foreign-body infections. Infect Immun. 77, 3978–3991 (2009). 19581398 10.1128/IAI.00616-09 PMC2738049

[b65] Arciola C. R. . Detection of biofilm formation in Staphylococcus epidermidis from implant infections. Comparison of a PCR-method that recognizes the presence of ica genes with two classic phenotypic methods. J Biomed Mater Res A. 76, 425–430 (2006). 16270350 10.1002/jbm.a.30552

[b66] Arciola C. R., Baldassarri L. & Montanaro L. Presence of icaA and icaD genes and slime production in a collection of staphylococcal strains from catheter-associated infections. J Clin Microbiol. 39, 2151–2156 (2001). 11376050 10.1128/JCM.39.6.2151-2156.2001 PMC88104

[b67] Conlon K. M., Humphreys H. & O’Gara J. P. icaR encodes a transcriptional repressor involved in environmental regulation of ica operon expression and biofilm formation in Staphylococcus epidermidis. J Bacteriol. 184, 4400–4408 (2002). 12142410 10.1128/JB.184.16.4400-4408.2002 PMC135245

[b68] Jeng W. Y. . Crystal structure of IcaR, a repressor of the TetR family implicated in biofilm formation in Staphylococcus epidermidis. Nucleic Acids Res. 36, 1567–1577 (2008). 18208836 10.1093/nar/gkm1176 PMC2275139

[b69] Zhu C. . Human beta-defensin 3 inhibits antibiotic-resistant Staphylococcus biofilm formation. J Surg Res. 183, 204–213 (2013). 23273885 10.1016/j.jss.2012.11.048

